# “V-PLASTY”: a novel technique to reconstruct pulmonary valvular and annular stenosis in patients with right ventricular outflow tract obstruction

**DOI:** 10.1186/1749-8090-8-55

**Published:** 2013-03-28

**Authors:** Hasim Ustunsoy, Gokhan Gokaslan, Ozerdem Ozcaliskan, Cem Atik, Osman Baspinar, Yavuz Arslanoglu, Eren Oral Kalbisade

**Affiliations:** 1 Department of Cardiovascular Surgery, Gaziantep University Medical Faculty, Gaziantep, Turkey; 2 Department of Pediatric Cardiology, Gaziantep University Medical Faculty, Gaziantep, Turkey

**Keywords:** Congenital heart disease, Tetralogy of fallot, Pulmonary valve stenosis

## Abstract

**Background:**

The goal of repair of right ventricular outflow tract obstruction with or without Tetralogy of Fallot (TOF) is to eliminate valvular and/or subvalvular obstruction. However, this operation has a high risk of late complication of pulmonary insufficiency. In this study, we aimed to present early period results of our new technique that we call “V-Plasty” developed to prevent pulmonary insufficiency after pulmonary valve reconstruction in selected patients.

**Methods:**

Between January 2006 and January 2010, we performed V-plasty for pulmonary valve reconstruction in 10 patients. Eight patients (5 males, 3 females) had TOF and 2 patients (1 male, 1 female) had atrial septal defect concomitant with pulmonary valve stenosis. Patient selection for V-plasty reconstruction was made due to the pulmonary valve anatomy and degree of stenosis. The mean follow-up time was 55.7 ± 16.2 months (ranging from 32 to 80 months).

**Results:**

Functional capacity of the patients improved immediately after the surgery. There were no mortality and re-operation in follow-up period. Patients were followed up with echocardiography one week after the operation, at 1st, 6th, 12th months and annually. There was no pulmonary insufficiency.

**Conclusions:**

Operative correction of the pulmonary outflow tract obstruction with or without TOF, frequently requires transannular enlargement because of the infundibular and/or annular-valvular obstruction. This conventional technique is usually a reason for late pulmonary insufficiency. In our study, we have not seen pulmonary insufficiency in early term follow-up period. Our early term results are encouraging, but long term follow-up results are needed with large case series.

## Background

Right ventricular outflow tract (RVOT) reconstruction had always become a challenge in congenital heart diseases for the surgeons. Since Rastelli first described surgical repair for pulmonary valve [1], surgeons performed various techniques up to anatomic situation of the RVOT and pulmonary valve. In all these surgical approaches preventing the pulmonary insufficiency has become the key point. Turrentine et al. reported that patients who only underwent to transannular patch enlargement without valve-sparing exhibits early perioperative morbidity which is manifest by right ventricle dysfunction, low cardiac output, and inotrope dependency causing prolonged ventilation requirements and intensive care unit (ICU) admissions [2]. Therefore, transannular enlargement of pulmonary annulus alone may not be sufficient and pulmonary valve sparing may be necessary to prevent complications related to pulmonary insufficiency such as right ventricle dysfunction and arrhythmia. Thus we aimed to reconstruct the RVOT obstruction with enlarging the anterior leaflet and pulmonary annulus with a patch while preserving its coaptation surface which will grow up with the patient’s age and will preserve native valve coaptation. In this study we present early term results of our new technique that we call “V-Plasty” to reconstruct pulmonary valve and enlarge pulmonary annulus in patients with mild or moderate pulmonary valvular and annular stenosis.

## Methods

**Figure 1:**
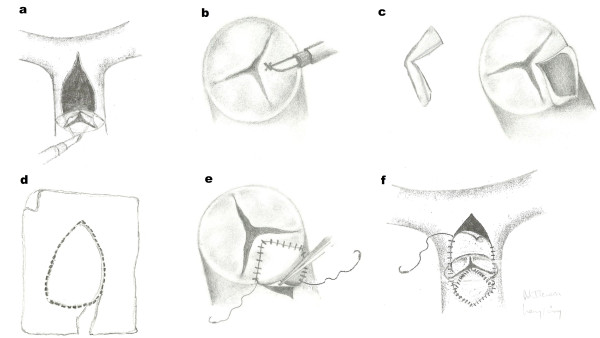
**Schematic view of the V-Plasty Technique. a- **General view of the pulmonary arteriotomy. **b- **Anterior leaflet incision starting point. **c- **Anterior leaflet of the pulmonic valve is incised longitudinally in midcusp line from 1-2 mm below the native free margin to the pulmonary annulus. After the incision, incised anterior leaflet forms a V shape. **d- **Shape of the pericardial patch which will be used for enlargement of the anterior leaflet. **e- **Annulus and anterior leaflet enlargement achieved with a pericardial patch. Suturing of the patch started at the level of the pulmonary annulus and continued through the free margin of the anterior leaflet. **f- **Construction of a new pulmonary sinus over anterior leaflet with a second patch which is bigger than the first one.

Institutional Review Board approval was obtained for the conduct of this retrospective study. Between January 2006 and January 2010, we performed V-plasty pulmonary valve reconstruction in 10 patients. We entitled this technique as “V-plasty” because anterior pulmonary leaflet forms a V shape after the incision for pulmonary annular patch enlargement (Figure 1c). The mean age of the patients was 5.2 (ranging from 2 to 11). Eight patients (5 males, 3 females) had tetrology of Fallot (TOF) and 2 patients (1 male, 1 female) had atrial septal defect concomitant with pulmonary valve stenosis. Demographic data of the patients were listed in Table 1. Dysplastic and bicuspid valves were excluded because these valve anomalies are not inappropriate to reconstruct with V-Plasty. Therefore, reconstruction with V-Plasty was performed for only mild and moderate annular – valvular stenosis with tricuspid pulmonary valve anatomy. In these patients also infundibular stenosis relieved with muscle band resection and patch augmentation. Ethic aproval was obtained from Gaziantep Universty clinical investigations ethic committee with reference number 22.11.2011/239.

**Table 1:** Demographic data of the patients

**Patient**	**Age (Years)**	**Gender**	**BSA(m**^**2**^**)**	**Pathology**
1	3	M	0,59	TOF
2	2	M	0,51	TOF
3	2	F	0,48	TOF
4	4	M	0,61	TOF
5	11	M	1,34	HVASD + PAPVC + Valvar PS
6	8	F	0,91	Secundum ASD + Valvar and Infundibular PS
7	3	F	0,53	TOF
8	8	M	0,98	TOF
9	5	F	0,63	TOF
10	6	M	0,68	TOF

*ASD *Atrial Septal Defect*, BSA Body Surface Area, F* Female*, HVASD* High Venosum Atrial Septal Defect*, M *Male*, TOF *Tetralogy of Fallot*, PS *Pulmonary Stenosis.

All procedures were performed with transesophageal echocardiography guidance.

### Operative technique

All surgical procedures were performed through median sternotomy. Aortic cannulation, selective vena cava superior and vena cava inferior cannulations were achieved. An aortic cross-clamp was applied and antegrade cold cardioplegia was administered into the aortic root to achieve prompt diastolic cardiac arrest. Snaring of vena cava superior and inferior, infundibular muscle bands were resected and ventricular or atrial septal defects were closed through the right atrium. Then, the pulmonary trunk was opened from 2–3 mm above of the pulmonary commissures. Orientation of commissures and the anterior (nonseptal) cusp was examined whether anterior leaflet was proper for our new reconstruction technique (Figure 1a). Anterior leaflet of the pulmonic valve was incised longitudinally in midcusp line from 1–2 mm below of the native free margin to the pulmonary annulus (Figure 1b). The incision was extended to below the annulus to provide adequate enlargement of the RVOT (Figure 1c). Enlargement of the pulmonary annulus was controlled passing a hegar bougie which was calculated according to the body surface area (Table 2). Bovine pericardium was used as patch. Patch size to enlarge anterior leaflet and RVOT was estimated with using the hegar bougie. Suturing of the patch with 6–0 polypropylene was started at the level of the pulmonary annulus and continued through the free margin of the pulmonary anterior leaflet (Figure 1d). After construction of new pulmonary annulus and anterior leaflet, the pulmonary arteriotomy was closed for construction of a new pulmonary sinus over anterior leaflet with a second patch which was bigger than the first one (Figure 1e).

**Table 2:** Appropriate pulmonary annular size according to BSA

**Pulmonary annulus (mm)**	**BSA (m**^**2**^**)**
8.4	0.25
9.3	0.30
10.1	0.35
10.7	0.40
11.3	0.45
11.9	0.50
12.8	0.60
13.5	0.70
14.2	0.80
14.8	0.90
15.3	1.0
16.2	1.2
17.0	1.4
17.6	1.6
18.2	1.8
18.0	2.0

*BSA *Body Surface Area.

#### Statistical analysis

Data are expressed as absolute values, percentages, or mean ± SD where appropriate.

## Results

The mean follow-up time was 55.7 ± 16.2 months (ranging from 32 to 80 months). Mean cross clamp time was 78.92 ± 12.85 minutes and mean cardio pulmonary bypass time was 122.74 ± 15.62 minutes. There were no early and late mortality and re-operation in our V-Plasty patients. We did not observe any rhythm disturbances.. Mean intubation time was 4.7 hours. Mean intensive care unit and hospital stay were 18.3 hours and 5.8 days, respectively. Patients were followed up with echocardiography by a single pediatric cardiologist one week after the operation, at 1st, 6th, 12th months and annually. Echocardiographic follow-up data was shown in Table 3. To determine whether RVOT enlargement was enough, intraoperatively measured pRV/pLV should be smaller than 0.70 [3-6]. All of our pRV/pLV measurements were smaller than 0,70. Functional capacities of the patients improved immediately after the surgery.

**Table 3:** Effects of V-Plasty on the pulmonary valve function and RVOT Gradient

**Patient**	**BSA (m**^**2**^**)**	**Diameter of Hegar Bougie(mm)**	**Preoperative RVOT Gradient (mmHg)**	**Postoperative RVOT Gradient (mmHg)**					**Postoperative PI**					**pRV/pLV**
				**1**^**st **^**Week**	**1**^**st **^**Month**	**6**^**th **^**Month**	**12**^**th **^**Month**	**Last follow-up**	**1**^**st **^**Week**	**1**^**st **^**Month**	**6**^**th **^**Month**	**12**^**th **^**Month**	**Last follow-up**	
**1**	0,59	12,5	88	12	14	12	15	18	Mild	Mild	Mild	Mild	Mild	0,56
**2**	0,51	12	76	8	9	10	8	11	Mild	Mild	Mild	Mild	Mild	0,52
**3**	0,48	12	74	5	5	5	8	10	None	None	None	None	Mild	0,57
**4**	0,61	13	57	13	15	15	12	12	Mild	Mild	Mild	Mild	Mild	0,45
**5**	1,34	16,5	77	6	8	5	8	10	Mild	Mild	Mild	Mild	Mild	0,42
**6**	0,91	15	81	10	10	12	10	12	None	None	None	None	None	0,48
**7**	0,53	12	66	10	8	10	12	15	Mild	Mild	Mild	Mild	Mild	0,55
**8**	0,98	15	63	15	15	16	10	12	None	None	None	None	None	0,56
**9**	0,63	13	75	13	15	18	14	14	None	None	None	None	None	0,64
**10**	0,68	13,5	62	11	10	10	10	8	None	None	None	None	None	0,47

*BSA *Body Surface Area*, RVOT, *Right Ventricular Outflow Tract*, PI* Pulmonary Insufficiency*, pRV *Right Ventricle Pressure*, pLV *Left Ventricle Pressure*.*

## Discussion

The operative repair of RVOT obstruction generally requires either resection of the dysplastic valve or widening of the pulmonary valve annulus using a transannular patch. However patch augmentation of pulmonary valve alone leads to pulmonary insufficiency which causes right ventricle volume overload, right ventricle failure and fatal arrhythmias. Borowski and colleagues reported that pulmonary insufficiency develops in up to 30% of patients at a follow-up of 20 years [7]. Therefore, a lot of various techniques were performed and developed since Kirklin first described transannular reconstruction in 1959 [8]. Rastelli first described repair of pulmonary valve in 1965 [1]. Then, various types of patch materials were used for reconstituting blood flow from the right ventricle to the pulmonary artery such as autologous pericardium, Dacron polyester fabric, polytetrafluoroethylene (PTFE), glutaraldehydetreated bovine pericardium, bovine jugular vein, cryopreserved allografts or homografts, and engineered tissue grafts. Each of these materials has substantial advantages and disadvantages including stenosis, thromboembolization, calcium deposition, and risk of infection [9-12]. We preferred bovine pericardium instead of autologous pericardium in our cases due to its easier application. At the same time, native pericardium was spared to close mediastinum to reach heart easily when a re-operation necessitated.

On the other hand, the nonvalved conduit without a pulmonary valve is not physiological and this contributes to the occurrence of postoperative mortality and morbidity [13]. Besides, pericardial monocusp or homograft monocusp reconstruction to provide pulmonary coaptation, valve function decreases over time as the RVOT grows and the homograft tissue undergoes structural deterioration [14]. Therefore, we suggested that creation of a functional pulmonary valve is the key point of the RVOT reconstruction in proper patients. In our study, we performed the surgical technique of enlargement of the pulmonary annulus and the anterior pulmonary leaflet with a transannular patch of bovine pericardium in 10 consecutive patients who had non-dysplastic valve structure and mild or moderate pulmonary valvular/annular stenosis. The major key points of this technique are preserving pulmonary anterior leaflet coaptation surface while performing annulus enlargement, minimized surgical incision to preserve infundibulum function, infundibular muscle band resection to relieve RVOT stenosis, infundibular augmentation and construction of a new pulmonary sinus with a pericardial patch after enlargement of the anterior leaflet and annulus. The most important advantage of this technique is preserving the native valvular coaptation surface which will grow up with the patient’s age.

## Conclusions

In our follow-up period we have not seen neither moderate nor severe pulmonary insufficiency in our selected patient group. Our early results are encouraging, but long term follow-up results are needed with large case series.

## Competing interests

The authors declare that they have no competing interests.

## Authors’ contributions

U H and G G carried out the study design and drafted the manuscript, O O, A Cem and B O collected patients’ data, A Y and K E O participated in the design of the study. All authors read and approved the final manuscript.
